# Extracellular Matrix: A Treasure Trove in Ovarian Cancer Dissemination and Chemotherapeutic Resistance

**DOI:** 10.7759/cureus.13864

**Published:** 2021-03-13

**Authors:** Surbhi Valmiki, Mohamed A Aid, Ali R Chaitou, Maria Zahid, Mrinaal Valmiki, Peter Fawzy, Safeera Khan

**Affiliations:** 1 Obstetrics and Gynecology, California Institute of Behavioral Neurosciences & Psychology, Fairfield, USA; 2 Intensive Care Unit, California Institute of Behavioral Neurosciences & Psychology, Fairfield, USA; 3 Intensive Care Unit, King Fahad Military Medical Complex, Jeddah, SAU; 4 Internal Medicine, California Institute of Behavioral Neurosciences & Psychology, Fairfield, USA; 5 Internal Medicine, Faculty of Medical Sciences, Lebanese University, Beirut, LBN; 6 Psychiatry, California Institute of Behavioral Neurosciences & Psychology, Fairfield, USA; 7 Neurological Surgery, California Institute of Behavioral Neurosciences & Psychology, Fairfield, USA

**Keywords:** collagen, ovarian cancer, extracellular matrix, drug resistance

## Abstract

Late presentation and resistance to chemotherapeutic agents make a deadly combination for ovarian cancer patients. The treatment of these patients is thus challenging. This study explores the possible molecular mechanisms by which tumor cells interact with the extracellular matrix (ECM) constituents, forming metastatic implants and enhancing patients' sensitivity to drugs. For the literature review, PubMed was used as a database. The standard search was done using keywords "collagen, ovarian cancer, extracellular matrix, drug resistance" in different combinations, which finally yielded 32 studies meeting the inclusion/exclusion criteria. The studies included were published in the English language in the past seven years.

After analyzing, we found all of them to be histopathological studies. Nine studies also used murine cell lines besides human cell lines and tissue samples from ovarian cancer patients. One study has a retrospective analysis done. Eight studies demonstrate the role of hypoxia and matrix remodeling enzymes in ovarian cancer dissemination. Genetics playing a crucial role in cancer metastasis is demonstrated in eight studies. Ten studies included shows receptors, enzymes, and spheroid organization in disease progression. Six studies address chemotherapeutic resistance. Intraperitoneal dissemination of ovarian cancer and the development of chemotherapeutic resistance depends on certain molecular interactions, and they can be targeted to improve patients' overall survival.

## Introduction and background

Ovarian cancer remains the most lethal of all gynecological cancers, with approximately 14,000 deaths each year in the United States [1]. The Annual Incidence reported in the year 2018 was 22,240 [1]. Ovarian cancer patients are often asymptomatic and are not recognized until late in the disease course. The patients usually present with distant metastasis and malignant ascites hurting the overall survival. The patients initially respond to debulking surgery and chemotherapeutic regimen usually consisting of a platinum complex (cisplatin or carboplatin) and a taxane (paclitaxel or docetaxel), nearly 80% eventually relapse into chemo-resistant cases resulting in a five-year survival rate of 30% [2].

Unlike most solid tumors, which spread via lymphatic or hematogenous routes, ovarian cancer cells disseminate through direct extension into the peritoneum by shedding either as a single cell or multicellular aggregates (MCA), which interact with mesothelial cells lining the peritoneum, later invading the underlying basement membrane and spreading across the extracellular matrix (ECM) forming metastatic implants [3]. The extracellular matrix is mainly composed of collagen, laminin, fibronectin, vitronectin, proteoglycans, and gelatin, which also play a vital role in tumor invasion.

Cancer metastasis is a key component driving disease progression and patient survival, but the molecular mechanisms underlying it remains understudied. Previous studies described the role of hypoxia and hypoxia-inducible factor-α (HIF-α) in ovarian cancer proliferation and invasion [4,5]. Zhao et al. uncovered the role of an angiotensin receptor blocker (AT-1 receptor blocker) Losartan in decreasing the proliferation of ECM protein collagen, thereby leading to lesser drug resistance and decreased ascites in patients [6]. Several pathways (Janus kinase and signal transducer and activator of transcription proteins JAK/STAT3, mitogen-activated protein kinase-7 MAPK-7) [7,8], cell surface receptors like integrins [9], proteins such as microfibril-associated protein-5 (MFAP-5) [10], Tau protein [11], collagen triple helix repeat containing-1 (CTHRC-1) [12], receptors such as discoidin domain receptor-2 (DDR-2) [13], formyl peptide receptor type 1 (FPR1) [14], cytokines like transforming growth factor-β (TGF-β) [15], enzymes such as lysyl oxidase (LOX) [4], integrin-linked kinase (ILK) [16], and histone deacetylase-4 (HDAC-4) have been well characterized in the literature [17]. Glucocorticoids have also been shown to be associated with tumor cell adhesion and resistance to chemotherapeutic agents [18]. Further knowledge regarding the tumor-stromal interactions in the tumor microenvironment could help physicians sort preventive measures to decrease the morbidity and mortality associated with the disease.

Despite all the studies, little is known regarding the molecular mechanisms of tumor cell-mesenchymal cell interactions underlying metastasis. The studies described in this review mainly address the studies that have been done on tissue samples obtained from post-operative patients with ovarian cancer or the cell lines purchased from repositories. Nevertheless, the phenomenon of tumor seeding onto the peritoneum and peritoneal organs causing bowel obstruction, ascites leading to impaired circulation, and impaired drug delivery need further exploration in in-vivo experiments on human subjects.

The following research paper aims to review all the possible mechanisms of tumor proliferation, invasion, and metastasis, the role of ECM in intraperitoneal dissemination of tumor cells, and enhancing the sensitivity to chemotherapeutic agents discovered in the year 2013-2020. Our study reviews the role of the tumor microenvironment, receptors, pathways/regulators that may serve as therapeutic targets in the disease progression of ovarian cancer patients.

## Review

Methods

This traditional review was done without following PRISMA guidelines. The PRISMA flow diagram was however, included to explain the search strategy.

Search Strategy

A detailed literature review was done using the PubMed database for studies published from January 2013 to November 2020. The search for the studies was done manually using the regular keywords "collagen AND ovarian cancer, extracellular matrix AND ovarian cancer, drug resistance AND ovarian cancer".

Study Records

The relevant clinical, pathological, and pathophysiological data was stored and organized in a word document in Microsoft Word. An independent reviewer (SV) screened the studies using the title and abstracts based on eligibility criteria and relevance to the research question. In case of uncertainty, full articles were reviewed to determine the eligibility for inclusion and framed into this traditional review.

Ethical Issue

All the data in the following article was collected ethically and legally. All the included studies in this review had full-text links available freely on PubMed.

Inclusion/Exclusion Criteria

Types of patients and conditions: We reviewed the studies that included tissue samples from women of any age who suffered from ovarian cancer. The reviewed study also includes cell lines prepared in the laboratory or obtained from repositories.

Types of outcomes: We searched for the mechanism underlying distant metastasis in ovarian cancer patients, which is the major reason for patients' suffering and decreased survival, the mechanisms responsible for chemotherapeutic resistance to the drugs to improve the sensitivity for a better prognosis and survival.

Types of studies: Mixed human studies relevant to the research question published in peer-reviewed journals were included. There were no geographical restrictions, and articles published only in the English language were included.

Results

**Figure 1:**
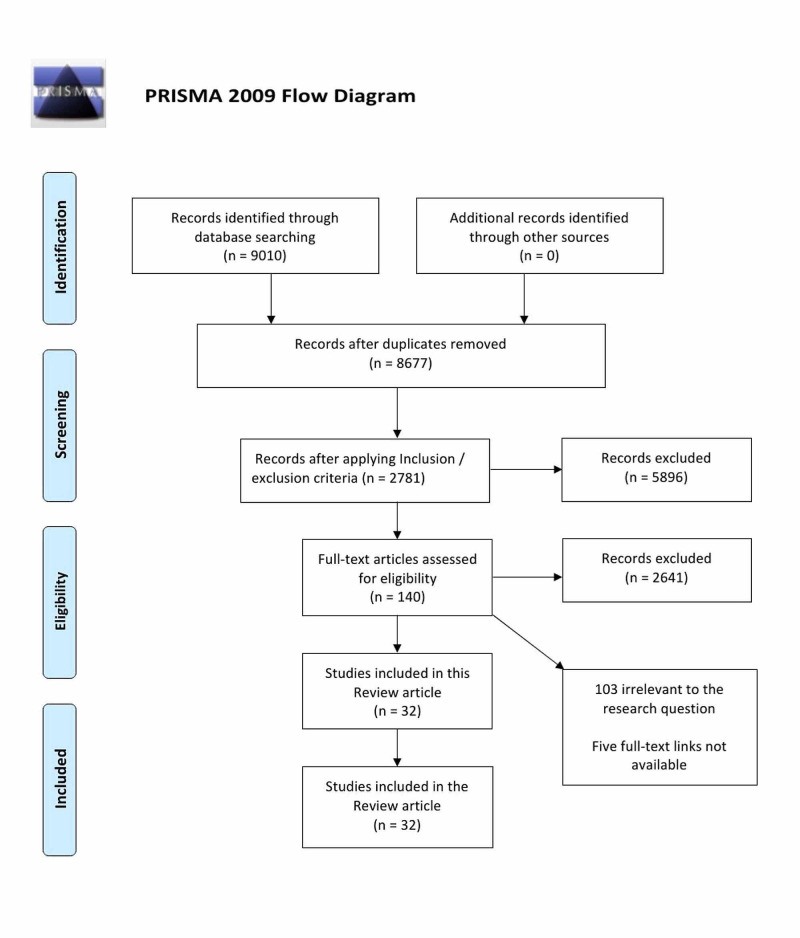
Preferred Reporting Items for Systematic Review and Meta-Analysis (PRISMA) Flow Diagram

Using the PubMed database, a literature search was done with four keywords, which yielded 9010 studies; after removing 333 duplicates using Mendeley citation manager, 8677 studies were left. On applying inclusion/exclusion criteria, 2781 articles remained, which were screened using title and abstracts. As a result, 2641 studies were excluded. One hundred and forty studies were left. Full-text articles were assessed, which led to the exclusion of 103 due to non-relevance to the research question, and five studies were excluded due to the unavailability of full-text links. In this review, 32 studies were finally included. The number of studies selected with each keyword is collagen (seven studies), ovarian cancer (14 studies), extracellular matrix (five studies), drug resistance (six studies), making a total of 32 studies [4-35]. Figure 1 below represents the Preferred Reporting Items for Systematic Review and Meta-Analysis (PRISMA) flow diagram.

Thirty-two histopathological studies assessing the role of extracellular matrix in ovarian cancer dissemination using a total of 672 female patient's tissue samples, 103 human cell lines, and three murine cell lines were studied. Peritoneal xenografts were used in three studies.

Study characteristics: A total of 32 studies were included, from which information was extracted. A summary of the involved studies is given in Table 1[4-35].

**Table 1:** Synopsis of Involved Studies ACEIs: angiotensin-converting enzyme inhibitors, ARBs: angiotensin receptor blockers, ANOVA: analysis of variance, MTT: 3-[4, 5-dimethylthiazol-2-yl]-2, 5-diphenyl tetrazolium bromide, CAM: chorioallantoic membrane, RT-PCR: real-time polymerase chain reaction

Reference	Study characteristics	Patients/tissue samples/cell lines	Tests performed	Statistical analysis
Natarajan et al. 2019 [4]	Histopathological study; Animal study	High grade serous ovarian cancer cell line: OVCAR5, OVCAR8 Human peritoneal mesothelial cell line: LP-9 Primary human mesothelial cells (PHMC): omentum of patients with benign disease Mouse ovarian cancer cell line: ID8	Gene signature analysis, siRNA analysis, collagen gels, and confocal microscopy, invasion assay, real-time quantitative polymerase chain reaction (qPCR), Western blotting, Immunohistochemistry, immunofluorescence, second harmonic generation microscopy, picrosirius red staining	It was done with two-way ANOVA and a two-tailed unpaired t-test. Statistical significance of p-value <0.05.
Yeung et al. 2019 [10]	Histopathological study; Animal study	OVCAR432 cells	IVIS bioluminescence fluorescence imaging system	It was done with a two-tailed unpaired Student’s t-test—the statistical significance of p-value <0.05.
Zhao et al. 2019 [6]	Retrospective analysis; Animal study	One hundred and twenty-three patients with stage IIIC or IV who were previously taking ACEIs or ARBs compared to 99 patients taking other anti-hypertensives SKOV3ip1, HeyA8	Gaussia Luciferase measurement, extravasation of doxorubicin by histology, planar-cut method to measure solid stress, unconfined compression test to quantify Young's modulus (stiffness), lymphatic vessel drainage function study, histology and immunohistochemistry, mRNA, miRNA extraction and array analysis, transient transfections and reporter gene assays	It was done using logistic regression. It was done with the Student’s t-test (two-tailed) or Mann-Whitney U test (two-tailed), two-sided Fisher's exact test.
Grither et al. 2018 [13]	Histopathological study	Patient-derived tumor cells–ascites from patients with ovarian cancer (POV) 1,9,10,12 Established human ovarian tumor cell lines—A2780, SKOV3ip1, OVCAR3, OVCAR5	Western blot analysis, gelatin zymography, immunohistochemical analysis using human tissue microarrays, Invasion and migration assays, proliferation assay, real-time polymerase chain reaction (PCR) with reverse transcription, fibronectin cleavage assay, cell spreading assay, spheroid-induced mesothelial clearance assay, survival analysis.	It was done using a two-tailed unpaired Student’s t-test with a statistical significance of p-value <0.05.
Guo et al. 2017 [12]	Histopathological study; Animal study	Tissue samples from 72 patients of primary epithelial ovarian cancer (PEOC)- 34 out of 72 were stage I-II, and the remaining 38 patients were stage III-IV. Ten normal tissue samples. Human ovarian adenocarcinoma-ascites of a 64-year-old woman: SKOV3 Human ovarian clear cell carcinoma: ES2 Ovarian adenocarcinoma: A2780 Ovarian serous adenocarcinoma-ascites of Chinese patients: HO8910 Immortalized ovarian surface superficial epithelium cell line: IOSE	Transwell migration and invasion assays, wound healing assays, cell adhesion assays, phospho-antibody microarray, Western blotting, Co-immunoprecipitation, RNA extraction, and real-time RT-PCR assays, immunohistochemistry	It was done with a double-sided Student’s t-test and Chi-square test. Statistical significance of p-value <0.05.
Fogg et al. 2019 [7]	Histopathological study	High grade serous ovarian cancer (HGSOC) cell lines: OVCAR3, OV90, OVCA433	Polymerase chain reaction (PCR), histological analysis, confocal imaging, and image analysis	Done using one-way ANOVA, two-way ANOVA, or t-test
Chan et al. 2016 [25]	Histopathological study	HEYA8, OVCAR8	Scratch wound invasion assay, scanning electron microscopy imaging, mRNA/miR isolation, and quantitative polymerase chain reaction, predicted miR targeting, Parallel microfiltration, Microfluidic device fabrication, and operation using standard soft lithography, Flow cytometry.	It was done separately for each assay with a Statistical significance of p-value <0.05.
Samardzija et al. 2016 [9]	Histopathological study; Animal study	Primary high-grade serous epithelial ovarian tumor and normal ovarian tissues from patients. Four established human epithelial ovarian cancer cell lines: SKOV3, OVCAR5, OVCA433, HEY	Immunofluorescence analysis, RNA extraction and real-time PCR, Western blotting, sphere forming assay, flow cytometric analysis, adhesion assay, gelatin zymography	It was done using two-way ANOVA and Dunnett's multiple comparison test with a Statistical significance of p-value <0.05.
Choi et al. 2016 [31]	Histopathological study	Primary human ovarian surface epithelial cell line: MCAS, OVCA432, OVCA433 HPVE6E7 immortalized OSE cell lines. Normal human ovarian surface epithelial (OSE) cell line: OSE7, OSE9	Immunofluorescence microscopy, immunohistochemical staining of tissue samples, gene expression profiling and network analysis, quantitative real-time reverse transcription PCR, Western blot analysis	It was done with ANOVA and a two-tailed t-test. Statistical significance of p-value <0.05.
Liu et al. 2019 [21]	Histopathological study	One hundred and forty paraffin-embedded tissue samples from patients after surgery. Out of 140, 60 were primary epithelial ovarian cancer, 30 were borderline ovarian tumors, 30 benign ovarian tumors, 20 normal ovarian tissues. Cell line- RMG-I-hFUT	Reverse transcription-quantitative polymerase chain reaction (RT-qPCR) analysis, Western blot analysis, co-immunoprecipitation assay, Confocal laser scanning microscopy, Cell adhesion assay, immunohistochemical staining, immunocytochemical staining	It was done with Student’s t-test, chi-square test, one-way analysis of variance with LSD or Bonferroni posthoc test, and Kaplan-Meier curves. Statistical significance of p-value <0.05.
Klymenko et al. 2017 [30]	Histopathological study	Epithelial ovarian carcinoma cell line: OvCa432, OvCa433, OvCa429, DOV13 Ovarian adenocarcinoma cell line: OVCAR3, SKOV3 Human mesothelial cell line: LP9	Quantitative polymerase chain reaction of cDNA arrays, Western blot analysis, dual-label immunofluorescence microscopy (DLIF), MCA and tissue scanning electron microscopy (SEM), transmission electron microscopy (TEM), cell proliferation assay, cell migration assay, Matrigel invasion assay, cell-to-collagen adhesion assay, cell-to-mesothelium adhesion assay, cell-to-peritoneum adhesion assay.	Done using Student's t-test
Klymenko et al. 2017 [22]	Histopathological study; Animal study	Epithelial ovarian cancer cell lines: OvCa433, DOV13 Human peritoneal mesothelial cell line: LP9 Murine epithelial ovarian cancer cell line: ID8	Cell adhesion assay, mesothelial clearance assay, scanning electron microscopy (SEM)	It was done using the two-sided Mann-Whitney U test with a Statistical significance of p-value <0.05.
Deng et al. 2017 [34]	Histopathological study	Human ovarian cancer cell line: SKOV3, HO-8910 Immortalized ovarian epithelial cell line: IOSE386	Immunohistochemistry, RNA extraction, and quantitative PCR, DNA methylation analysis, chromatin immunoprecipitation assay, wound healing assay, invasion assay, colony formation in soft agar, cell cytotoxicity assay, flow cytometric detection of apoptosis	It was done using a two-tailed Student’s t-test and Pearson's correlation test with a Statistical significance of p-value <0.05.
Ye et al. 2016 [26]	Histopathological study	Eighty-three tissue samples of human ovarian cancer with 18 normal ovarian tissue samples as controls Human ovarian cancer cell lines: SKOV3, ES2, CAOV3, HEY, COV318	Immunohistochemical staining, RNA interference-based gene silencing experiment, Western blot analysis, quantitative real-time PCR, cell viability assay, in-vitro migration, and invasion assay	Done using Pearson's test, Kaplan-Meier method, Student’s t-test with a statistical significance of p-value <0.05.
Shen et al. 2016 [17]	Histopathological study	Tissue samples of 102 patients with epithelial ovarian cancer Epithelial ovarian cancer cell lines: OVCAR3, SKOV3	Immunohistochemistry, immunofluorescence assay, immunoprecipitation assay, Western blot analysis, cell migration assay, quantitative qPCR	It was done using an unpaired Student’s t-test with a statistical significance of p-value <0.05.
Yin et al. 2016 [18]	Histopathological study	Human ovarian cancer cell line: SKOV-3, HO-8910	Real-time PCR, Western blot, enzyme-linked immunosorbent assay (ELISA), RNA interference, cell adhesion assay, cell viability assay	It was done using ANOVA with a statistical significance of p-value <0.05.
Klymenko et al. 2018 [29]	Histopathological study	Epithelial ovarian cancer (EOC) cell lines: OvCa429, OvCa433, DOV13, SKOV3	Analysis of proliferation, Western blot analysis, RNA extraction, and qPCR, scanning electron microscopy (SEM)	It was done using the two-sided Mann-Whitney U test with a statistical significance of p-value <0.05.
Bruney et al. 2016 [16]	Histopathological study	Ovarian cancer cell lines: DOV13, OVCA433, SKOV3, ES2 Human peritoneal mesothelial cell line: LP9	Western blotting and immunoprecipitation, quantitative real-time PCR (qPCR), Immunohistochemistry, immunofluorescence, adhesion, and invasion assay	Done using Student’s t-test, Mann-Whitney U test, Kruskal-Wallis test with a Statistical significance of p-value <0.05.
Price et al. 2020 [27]	Histopathological study; Animal study	Human ovarian cancer cell lines: OVCAR3, OVSAHO, OVCA429, A2780, SKOV3-IP1 Mouse cell line: ID8 IP2	Immunoblotting, RT-PCR, ID8 IP2 in vivo modeling Histology, flow cytometry, adhesion to ECM, migration scratch/wound assay, invasion assay, proliferation assay,	Done using two-tailed Welsh's t-test, Mental-Cox and Gehan-Breslow- Wilcoxon test with a statistical significance of p-value <0.05.
Bruney et al. 2014 [20]	Histopathological study	Ovarian cancer cell line: OVCA433 Ovarian adenocarcinoma cell line: OVCAR3 Human mesothelial cell line: LP9	Immunohistochemistry, immunofluorescence, quantitative real-time PCR (qPCR), florescence-activated cell sorting (FACS) analysis, enzyme-linked immunosorbent assay, mesothelial cell adhesion assay, meso-mimetic invasion assay, adhesion to peritoneal explant	Done using Student’s t-test
Li et al. 2020 [23]	Histopathological study	Human ovarian cancer epithelial cell line: OVCAR3 Human normal ovarian epithelial cell line: IOSE80	Quantitative reverse transcription PCR (qRT-PCR), cell proliferation activity assay, flow cytometry, transwell cell migration and invasion assay, double luciferase activity assay, Western blot	It was done using a t-test and analysis of variance (ANOVA) with a statistical significance of p-value <0.05.
Cheon et al. 2014 [15]	Histopathological study; Animal study	Snap-frozen and paraffin-embedded patient samples. Cancer cell lines: OVCAR3, A2780 Normal cell lines: TRS3	Microarray data analyses, validation of the 10-gene signature, RNA isolation and RT- qPCR analysis, molecular pathway analysis, in situ hybridization, immunohistochemistry	It was done using an unpaired t-test with a statistical significance of p-value <0.05.
Flate and Stalvey 2014 [28]	Histopathological study	Cisplatin sensitive cell line: OV2008 Cisplatin resistant cell line: C13	Wound healing assay, migration assay, Western blot analysis, microarray	It was done using one-way ANOVA, Tukey, and LSD post hoc tests with a statistical significance of p-value <0.05.
Dai et al. 2015 [8]	Histopathological study	Human ovarian cancer cell line: OVCAR-3	RT-PCR analysis, analysis of cell proliferation using MTT assay, cell migration assessment, cell invasion assay, Western blot	It was done using an independent sample t-test and Post-hoc Turkey-test with a statistical significance of p-value <0.05.
Sun et al. 2015 [19]	Histopathological study	Highly metastatic human ovarian cancer cell line: HO-8910PM	Three-dimensional type I collagen invasion and degradation assay, RT-PCR analysis, Western blotting analysis, cell surface biotinylation, fluorescent immunocytochemistry, CAM invasion assay.	N/A
Vallen et al. 2014 [33]	Histopathological study	Tissue samples from 25 patients of serous subtype of ovarian cancer Human ovarian cancer cell line: SKOV3	Immunohistochemistry, reverse phase-high performance liquid chromatography (RP-HPLC) disaccharide analysis, two-dimensional scratch assay, hanging drop experiment, cell migration assay	N/A
Pettee et al. 2014 [32]	Histopathological study	Human ovarian cancer cell lines: ES-2, SKOV3, OVCAR3, OVCAR4, OVCA420, OVCA429, OVCA194	Adhesion, migration and invasion assays, microscopy, Dunn chemotactic migration assay, spheroid formation, and collagen embedding, two-dimensional immunofluorescence (IF), three-dimensional Immunofluorescence and Invasion assay, RhoA GTPase G-LISA activation assay,	It was done using a one-tailed Student’s t-test with a statistical significance of p-value <0.05.
Gurler et al. 2015 [11]	Histopathological study	Normal and pathologic ovarian tissue samples: OV2161, OV2087, OV808 Human ovarian cancer cell lines: OVCAR-4, SKOV-3, OAW28, Kuramochi, OVSAHO, OVKATE	Immunohistochemistry, immunofluorescence staining, subcellular fractionation, Western blot, transient transfections, paclitaxel treatment, cell survival, and clonogenic assay	It was done with the Student’s t-test and the statistical significance of p-value <0.05.
Triulzi et al. 2014 [35]	Histopathological study; Animal study	Human ovarian cancer cell line: SKOV3	Proliferation and doxorubicin response assays, Western blotting, confocal microscopy, Immunohistochemical (IHC) analyses, RNA extraction, and quantitative real‐time PCR	It was done using a t-test, chi-square test, two‐tailed unpaired student's t‐test with a statistical significance of p-value <0.05.
Wahab et al. 2020 [24]	Histopathological study	Twenty-four unmatched snap-frozen ovarian tissue samples from patients consisting of 11 serous ovarian cancer and 13 normal ovarian tissues Ovarian adenocarcinoma cell lines: Caov3 and ovarian adenocarcinoma, ascites cell line: SKOV3	MicroRNA expression profiling and validation, bioinformatics analysis, quantitative real-time PCR, cell viability, migration, and invasion assays	It was done using the Kruskal-Wallis and LIMMA statistical tests, Student’s t-test with a statistical significance of p-value <0.05.
Minopoli et al. 2019 [14]	Histopathological study	Tissue samples of 42 patients with different subtypes of epithelial ovarian cancer Human ovarian carcinoma cell line: SKOV-3, A2780	Tissue microarray building, Immunohistochemistry, Flow cytometry, the culture of mesothelial cells, Western blot, adhesion of epithelial ovarian cancer (EOC) cells onto extracellular matrix proteins, adhesion and invasion assay, fluorescence microscopy, ligand binding assay.	Done using one-way ANOVA and post hoc Dunnett t-test, Pearson's chi-square test with a statistical significance of p-value <0.05.
Zhang et al. 2019 [5]	Histopathological study	Ninety-one formalin-fixed and paraffin-embedded tissue samples from patients with high-grade serous adenocarcinoma Ovarian cancer cell line: A2780	Immunohistochemistry, Western blot analysis, RT-PCR, transwell migration assay	Done with unpaired Student’s t-test, Pearson's correlation analysis. Statistical significance of p-value <0.05.

Discussion

**Figure 2:**
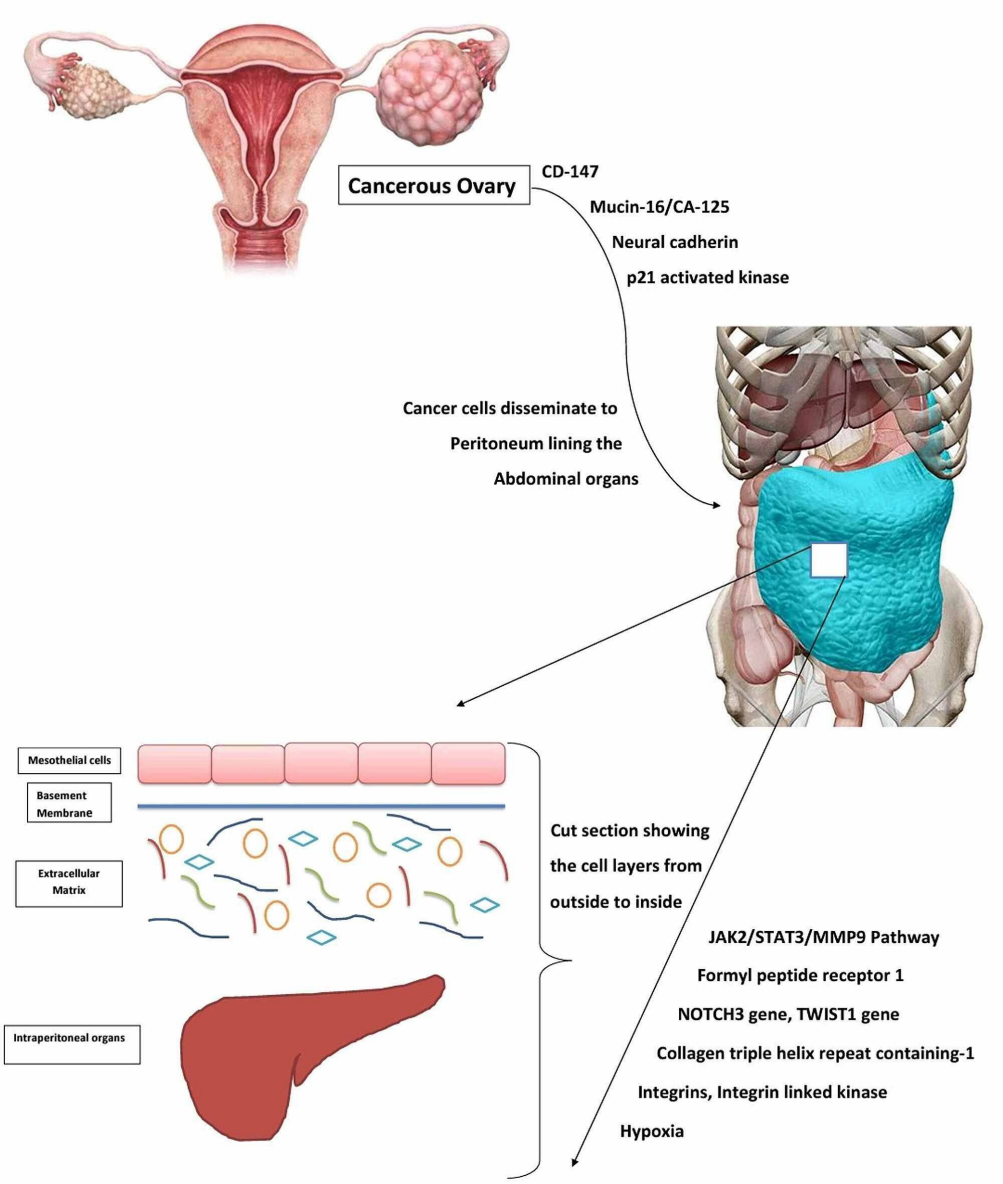
Pathophysiology of Ovarian Cancer Metastasis

Early metastasis and late presentation of the patients with metastasis to the peritoneum, causing ascites, is a distinct feature of ovarian cancer. Initial response to chemotherapy and later development of resistance is another feature of ovarian cancer. The molecular mechanism underlying these processes may be targeted to prevent tumor spread. Figure 2** **shows a schematic view of the pathophysiology of ovarian cancer metastasis [4,5,7,9,12-14,16,18-23,26-28].

Hypoxic Tumor Microenvironment and Matrix Metalloproteinases

Hypoxia has been shown to promote ovarian cancer cell invasion. Natarajan et al. showed enhanced expression of proline-lysine hydroxylases and lysyl oxidase in mesothelial cells leading to increased collagen deposition and increased collagen cross-linking respectively in the hypoxic tumor microenvironment, and this collagen remodeling subsequently leads to tumor invasion as hypoxia-inducible factor-1 and factor-2 (HIF-1 and 2) gets stabilized [4]. Zhang et al. showed increased mRNA and protein levels of HIF-α and matrix metalloproteinase-13 (MMP-13) in response to hypoxia, both of which decreased after transfection with small interfering RNA (siRNA) for HIFα. Matrix metalloproteinases are enzymes responsible for degrading ECM proteins, making tumor cells invade [5]. In contrast, Sun et al. showed hypoxia-induced ovarian cancer cell invasion using hydroxyproline levels to detect collagen degradation by membrane-type 1-matrix metalloproteinase (MT1-MMP). They also showed increased mRNA and protein levels of MT1-MMP in response to hypoxia, which aids in invasions like Zhang et al. Hypoxia also causes increased Snail (a zinc-finger transcription factor) expression, which leads to enhanced expression of MT1-MMP responsible for three-dimensional collagen invasion [19].

The glycoproteins expressed on the surface of tumor cells mediate cell adhesion and subsequent invasion. CD147, a highly glycosylated protein on the tumor cell surface, having Lewis antigen on its surface promotes CD147 mediated tumor cell adhesion and expression of matrix metalloproteinase-2 (MMP-2). CD147 and Lewis-Y antigen expression was associated with a higher adhesive ability to ECM proteins collagen and laminin, higher tumor grade, metastasis to lymph nodes, and decreased survival [21]. Another glycoprotein, Mucin 16 (MUC 16)/cancer antigen (CA-125), expressed on tumor cells binds mesothelin of mesothelial cells of the peritoneum, which is the initial event in tumor adhesion and spreading across the ECM. Matrix metalloproteinases (MT1-MMP, MMP-14) were seen catalyzing the degradation of MUC-16/CA-125, eventually leading to its decreased surface expression, and attachment to peritoneum was decreased [20].

The cadherin composition and expression of matrix metalloproteinases (MMP) govern collagen matrix invasion and peritoneal metastasis. Klymenko et al. highlighted neural cadherin's (Ncad) presence on MCA to have significant depth penetration, rapid lateral migration through the mesothelium before disrupting the basement membrane to invade ECM compared to epithelial cadherin (Ecad) expressing cells. Inhibition of MMP showed decreased invasion of cancer cells [22]. Integrins play a role in the adhesion of tumor cells to ECM proteins. Oct4A is a transcription factor, suppression of which is associated with decreased expression of integrin β1, α5, and α2 subunits leading to diminished adhesion of tumor cells to collagen and fibronectin, decreased levels of MMP-2, and certain markers. Upregulation of Oct4A was linked to the formation of stable, highly compact MCA [9]. Another study showing the formation of more cohesive MCA was coupled with the expression of integrin-linked kinase (ILK), co-expressed with MT1-MMP. ILK catalyzes the phosphorylation of cytoplasmic tails of MMP, which leads to the formation of stable MCA, which causes metastasis. ILK also regulates Integrin-mediated cell adhesion causing mesothelial cells to retract, uncovering the ECM [16]. Table 2 below highlights the objectives and potential therapeutic targets of the included studies [4,5,9,16,19-22].

**Table 2:** Objectives and Potential Therapeutic Targets HIF-LOX: hypoxia-inducible factor-lysyl oxidase, MMP: matrix metallopeptidase, MCA: multicellular aggregates, MT1-MMP: membrane-type 1-matrix metalloproteinase, MUC-16: Mucin-16

Authors	Year of publication	Purpose of the study	Potential therapeutic target
Natarajan et al. [4]	2019	To demonstrate the role of hypoxia in the stabilization of HIF-1 and HIF-2 and increased expression of LOX leading to increased collagen deposition, cross-linking of collagen, and tumor cell invasion	HIF-LOX signaling axis
Zhang et al. [5]	2019	To illustrate the effect of hypoxia-induced HIF-1α on the expression of MMP-13 and ovarian cancer invasion	HIF-1α and MMP-13
Liu et al. [21]	2019	To study the function and mechanism of Lewis-Y antigen in CD147 mediated tumor cell adhesion and progression in ovarian cancer	Lewis Y antigen on CD147 cell surface, CD147
Klymenko et al. [22]	2017	To evaluate the effect of cadherin expression profile and cellular architecture in the form of single-cell or MCA on ovarian tumor cell invasion of sub-mesothelial matrix leading to metastasis	Neural cadherin (Ncad)
Samardzija et al. [9]	2016	To demonstrate the Interdependence in the expression of Oct4A, integrins, pro-MMP2 in adhesion and invasion of ovarian cancer	Knockdown of Oct4A (a member of a family of transcription factors)
Bruney et al. [16]	2016	To assess the function of Integrin-linked kinase (ILK) and its co-expression with MT1-MMP causing ovarian cancer metastasis	ILK inhibitor
Sun et al. [19]	2015	To study the role of hypoxia in Snail mediated expression of MT1-MMP and tumor cell invasion in collagen 3D models	HIF-1α, Snail, MT1-MMP
Bruney et al. [20]	2014	To study the potential correlation between MT1-MMP & Mucin-16 and the role of MT1-MMP dependent ectodomain shedding of Mucin-16 in ovarian tumor cell adhesion and invasion	Increased MT1-MMP mediated shedding of MUC-16 might help prevent tumor cell adhesion

The Theatrical Role of Genetics

MicroRNA expression is a well-known cause of improved survival in ovarian cancer patients. Li et al. illustrated miR-30b-3p targeting its gene collagen triple helix repeat containing 1 (CTHRC1) at 3'UTR region, decreasing the protein expression of CTHRC1, which hinders epithelial to mesenchymal transformation in ovarian cancer cells. miR-30b-3p is an anticancer gene, and its overexpression inhibits migration and invasion of ovarian cancer cells, suppresses proliferation, promotes apoptosis [23]. Wahab et al. discovered 48 MicroRNAs acting on their targeted genes inhibiting ovarian cancer cell migration and invasion. MicroRNAs were shown to correlate with ovarian cancer histological subtype, tumor stage, chemoresistance to drugs, cancer recurrence, and survival [24]. Another study showed the role of tumor suppressor MicroRNAs in the invasion of ovarian cancer. MicroRNA overexpression leads to increased filamentous actin (F-actin) levels, which decreases the invasive potential of cancer cells through collagen matrices. Increased cell size and reduced deformability were seen with tumor suppressor MicroRNA transfection, making it useful as bigger cells have lesser invasive potential, and less deformable cells take longer to transit [25].

CTHRC1 is a cancer-related gene involved in certain signaling molecules' phosphorylation, enhancing the migration and invasion of ovarian cancer. A study by Ye et al. observed decreased phosphorylation of epidermal growth factor receptor (EGFR), extracellular signal-regulated kinase 1/2 (ERK1/2), protein kinase (AKT), and reduced migration and invasion of ovarian cancer cells on silencing the CTHRC1. They also observed that the EGFR inhibitors blocked the effect of CTHRC1. Hence it was suggested that CTHRC1, by activating EGFR signaling, promotes metastasis in ovarian cancer, which is mediated through ERK1/2, PI3K/AKT [26]. CTHRC1 was also found to be increasing the expression of Integrinβ3 and phosphorylating focal adhesion kinase (FAK), which led to invasion and migration of ovarian cancer cells, enhanced adhesion to ECM proteins, peritoneal and lymph node metastasis [12].

Cheon et al.'s gene signature analysis identified 10 collagen remodeling genes regulated by transforming growth factor-β (TGFβ1) signaling. COL11A1 was among the genes discovered, which showed significantly higher expression in metastatic tumors than primary tumors. These genes were found to promote metastasis and decreased survival [15]. Identification of downstream targets upon activation of NOTCH3 Intracellular domain (NOTCH3IC) showed induction of genes encoding ECM proteins and adhesion molecules. Collagen and Integrin genes were identified to promote cancer cell attachment to the peritoneum, causing ascites leading to poor survival [27]. An epithelial to mesenchymal transcription factor TWIST1 induces the expression of a mesenchymal gene discoidin domain receptor 2 (DDR2), which recognizes collagen as its ligand. DDR2 controls ECM remodeling enzymes' expression, which could lead to enhanced migration and invasion of tumor cells, cleavage of fibronectin, mesothelial cell clearance facilitating metastasis, and decreased survival [13]. Table 3below highlights the objectives and potential therapeutic targets of the included studies [12,13,15,23-27].

**Table 3:** Objectives and Potential Therapeutic Targets CTHRC1: collagen triple helix repeat containing 1, DDR2: discoidin domain receptor 2, EGFR: epidermal growth factor receptor, TGFβ: transforming growth factor-β

Authors	Year of publication	Purpose of the study	Potential therapeutic target
Li et al. [23]	2020	To investigate the role of anticancer gene miR-30b-3p on the biological activity of ovarian cancer cells and its association with the CTHRC1 gene	MicroRNA miR-30 family
Wahab et al. [24]	2020	To study the effect of differential expression of microRNAs and their target genes in ovarian cancer growth, migration, and invasion	MicroRNA
Price et al. [27]	2020	To assess the effect of NOTCH3 signaling on tumor cell adhesion to the peritoneum, tumor cell proliferation, and patient survival	NOTCH3
Grither et al. [13]	2018	To study the TWIST1 induced expression of DDR2 leading to mesothelial cell clearance and tumor cell invasion	DDR2
Guo et al. [12]	2017	To investigate the role of CTHRC1 in ovarian cancer cell migration, invasion, and adhesion to vitronectin, peritoneal metastasis, and metastasis to distant organs	CTHRC1, Integrin β3/FAK signaling
Chan et al. [25]	2016	To elucidate the mechanistic role of tumor suppressor microRNAs in ovarian cancer invasion and cancer cell physical properties	MicroRNA
Ye et al. [26]	2016	To study the effect of CTHRC1 induced EGFR signaling on ovarian cancer cell migration and invasion	CTHRC1
Cheon et al. [15]	2014	To identify collagen remodeling genes regulated by TGFβ signaling, promoting metastasis and leading to poor clinical outcome	Collagen remodeling genes, e.g., COL11A1

Metastatic Cascade: Receptors, Enzymes, and Spheroids Explained

Interaction of receptors was shown to increase ovarian cancer dissemination. Urokinase plasminogen activator receptor (uPAR) interacts with formyl peptide receptor 1 (FPR1) and promotes tumor cell adhesion to mesothelial cells of peritoneum and vitronectin. Higher expression of uPAR and FPR1 was correlated with metastasis and poor clinical outcome [14].

In addition to receptors, enzymes also promote ovarian cancer proliferation and dissemination. Shen et al. showed how histone deacetylase-4 (HDCA-4) enhanced cancer cell proliferation. Higher expression of HDCA-4 corresponds to a higher stage of cancer. HDCA-4 gets co-localized in the nucleus and PP1 in response to collagen matrices, which leads to altered transcription and translation of p21, promoting proliferation and migration of cancer cells [17]. Another study by Dai et al. showed mitogen-activated protein kinase 7 (MAPK7) associated with ovarian cancer cell proliferation, migration, and invasion. Type II collagen expression also increased with MAPK7 overexpression [8]. Flate and Stalvey showed that tumor cells interact with ECM proteins like collagen and fibronectin, which affects cell migration and invasion. They also highlighted p21 activated kinase (PAK) mediating the process of migration of cancer cells [28].

Cancer cells form spheroids by making cell-to-cell contact, affecting ovarian cancer cell behavior in the tumor microenvironment. Fogg et al., in their study, identified alternatively activated macrophages (AAM) in the ascitic fluid to be secreting certain soluble factors, e.g., Fms related tyrosine kinase 3 (FLT3), interleukin-2 (IL-2), interleukin-8 (IL-8), leptin, heparin binding-epidermal growth factor-like factor (HB-EGF). These factors were acting through a common pathway of JAK2/STAT3 activation, inducing MMP-9. This pathway leads to disaggregation of spheroids making single cells spread across the ECM [7]. Klymenko et al. showed increased lateral dispersion of spheroids (aka MCA) in response to ascites-induced compression. They also studied the effect of compression on MCA gene expression [29]. The expression of cadherins also influences metastatic success. Ncad+ cells form highly compact stable MCA, whereas Ecad+ cells tend to form loosely adherent cell clusters. Acquisition of Ncad by Ecad+ cells, making a hybrid increased migration, adhesion to mesothelial cells, invasion of ECM developing into secondary metastatic lesions [30]. Another study delineating the role of Cadherins showed high levels of E-cadherin in cancerous cysts. They also showed that E-cadherin repression led to cyst disruption and inhibited collective cancer cell migration. Hence, it was suggested that E-cadherin is important for cancer cells to migrate collectively in collagen matrices [31]. A study by Pettee et al. discovered a Rho GTPase effector mDia formin, which controls F-actin assembly required for cell-cell junction and tight spheroid formation. Inhibiting mDia led to the transition of cells acquiring an amoeboid configuration and invasive single cell dissemination. Rho-associated protein kinase (ROCK) is another Rho effector. Inhibiting both mDia and ROCK completely blocked invasion, which suggested that single-cell dissemination on inhibiting mDia is ROCK dependent [32]. Vallen et al. showed the overexpression of a 4,6 sulfated glycosaminoglycan (chondroitin sulfate E aka CSE) enhanced the adhesiveness between cells, which is seen in the spheroid formation and cancer metastasis [33]. Table 4below highlights the objectives and potential therapeutic targets of the included studies [7,8,14,17,28-33].

**Table 4:** Objectives and Potential Therapeutic Targets uPAR-FPR1: urokinase plasminogen activator receptor-formyl peptide receptor 1, AAM: alternatively activated macrophages, MCA: multicellular aggregates, TGFβ: transforming growth factor-β, HDAC4: histone deacetylase 4, MAPK7: mitogen-activated protein kinase 7, PAK2: p21 activated kinase 2, CSE: chondroitin sulfate E

Authors	Year of publication	Purpose of the study	Potential therapeutic target
Minopoli et al. [14]	2019	To illustrate the role of FPR1 in adhesion to mesothelial cell layer and vitronectin leading to invasion of ovarian cancer cells	Inhibition of uPAR-FPR1 interaction
Fogg et al. [7]	2019	To identify the role of soluble factors secreted from AAM in spheroid spreading across the ECM	JAK/STAT3/MMP-9 pathway
Klymenko et al. [29]	2018	To study the effect of ascites induced compression on invasion, lateral dispersion, the proliferation of cells of MCA	Drugs to eliminate ascites
Klymenko et al. [30]	2017	To study the diverse cadherin expression affecting the behavior of ovarian cancer MCA and single-cell dissemination	Cadherin
Choi et al. [31]	2016	To assess the effect of loss of E-cadherin on inclusion cyst formation and collective cancer cell migration	Cadherin, miR-200, TGFβ signaling
Shen et al. [17]	2016	To elucidate the mechanism of action of HDAC4 on ovarian cancer cell proliferation and migration via repression of p21	Nuclear HDAC4
Dai et al. [8]	2015	To study the role of MAPK7 on ovarian cancer cell proliferation, migration, invasion	Inhibition of MAPK7
Flate and Stalvey [28]	2014	To demonstrate the role of PAK2 mediating the ovarian cancer cells interaction with collagen type I and fibronectin, causing metastasis	Inhibition of PAK2
Pettee et al. [32]	2014	To study the effect of multiple Rho-GTPase effectors (mDia2, ROCK) on ovarian cancer cell invasion	mDia2/ROCK signaling axis
Vallen et al. [33]	2014	To study the mechanistic role of CSE in providing adhesiveness to tumor cells forming spheroids and cell migration	Chondroitin sulfate E (CSE) rich motifs

Combating the Chemotherapeutic Resistance

The amount of collagen in the tumor microenvironment regulates the response to chemotherapeutic drugs. Yeung et al. showed that cancer-associated fibroblasts (CAFs) secretes microfibril associated protein 5 (MFAP5), which causes increased expression of fibrosis-related genes thus, increasing the amount of collagen, which together with decreasing microvessel stability (more leakiness of vessels) leads to hindrance in the delivery of drugs [10]. Another study showed improved response to chemotherapy in patients who were given Losartan along with the standard therapy. Losartan decreases the number of fibroblasts, up-regulates antifibrotic miRNA decreasing the ECM content (collagen type I levels), and thus reduce the solid stress, which improves drug delivery [6]. In another study, doxorubicin diffusion was impaired due to increased collagen accumulation in the tumor microenvironment due to decreased susceptibility to proteolytic enzymes caused by a serine protease inhibitor Maspin. Tumor growth was significantly inhibited when the anti-Maspin antibody was given along with doxorubicin compared to doxorubicin alone [35].

Resistance to cisplatin was found to be governed by a tumor suppressor microRNA. In 2017, Deng et al. showed decreased expression of miR-199a-3p led to enhanced Dicoidin domain receptor-1 (DDR1) expression, which was responsible for resistance to cisplatin [34]. Dexamethasone (DEX) enhances fibronectin (FN) expression and mucin 16, responsible for its pro adhesive, pro-survival effects and protects cancer cells from chemotherapeutic agents. Inhibition of fibronectin with FN-siRNA and mucin 16 with MUC-siRNA attenuated the effects [18]. Ovarian cancer cells up-regulate the expression of a Microtubule-associated protein, which causes resistance to paclitaxel. Tau protein binds with microtubules and competes with paclitaxel for the binding site. Thus, enhanced Tau protein expression in ovarian cancer leads to resistance [11]. Table 5 below highlights the objectives and potential therapeutic targets of the included studies [6,10,11,18,34,35].

**Table 5:** Objectives and Potential Therapeutic Targets MFAP: microfibril associated protein, DDR1: discoidin domain receptor-1, MUC1: Mucin antigen 1

Authors	Year of publication	Purpose of the study	Potential therapeutic target
Yeung et al. [10]	2019	To elucidate the effect of MFAP5 blockade on tumor growth, fibrosis, blood vessel stability, and chemotherapeutic response to drugs	MFAP5
Zhao et al. [6]	2019	To study the role of losartan in improving the chemotherapeutic efficacy of drugs and decreasing ascites	Combining Losartan with standard treatment
Deng et al. [34]	2017	To elucidate the role of microRNA miR-199a-3p/DDR1 pathway on tumor migration, invasion, and drug resistance	DDR1
Yin et al. [18]	2016	To assess the effect of dexamethasone (a glucocorticoid) on tumor cell adhesion, survival, and resistance to chemotherapy	Fibronectin receptors, MUC1
Gurler et al. [11]	2015	To assess the response to paclitaxel in ovarian tumors with increased Tau protein expression	Inhibition of Tau protein expression
Triulzi et al. [35]	2014	To study the role of Maspin in chemotherapeutic response to doxorubicin	Maspin

Strengths and Limitations

To the best of our knowledge, this is to date the only traditional review gathering all the information regarding molecular mechanisms underlying the ovarian cancer metastasis addressing the interaction between tumor cells, peritoneal mesothelial cells, and the extracellular matrix discovered in the last seven years published in the English language in PubMed. The details of how tumor cells spread are described in these studies; however, what happens to the tumor cell structure, any configurational change in the enzymes, receptors, and other signaling molecules involved is not explained. The structural component of metastasis can be a topic of research in the future. Tumor cell interaction with peritoneum is well described in this review, but how do tumor cells initially travel from ovary to peritoneum have not been detailed in the studies and can be researched upon. The studies included in this review are done on human tissue samples and cell lines. Animal models were also used. This warrants the need to corroborate the findings in randomized control trials.

## Conclusions

The signaling molecules and pathways encountered in disseminating ovarian cancer and drug response lie in the peritoneum and extracellular matrix. Tumor cells detach from their primitive location on the ovaries and travel through the matrix to reach metastatic sites such as the peritoneum. They interact with peritoneal mesothelial cells and establish new sites for tumor growth and proliferation. Based on the reviewed articles, we found a substantial relationship between hypoxia, matrix remodeling enzymes, and disease progression. Several genes, their mRNAs, and subsequent proteins have been identified to alter patients' overall survival. There is a cascade of enzymes, receptors working in harmony to promote metastasis. The emerging chemotherapeutic resistance imposes great difficulties in treating patients, and targeting these molecular mechanisms has shown an improved response to the therapy. There is no good screening method available for early diagnosis before the patient develops metastasis. Studies focusing on finding a suitable marker for screening should be done. If studied in detail with more experimental studies and possibly targeted, the mechanisms highlighted in this review may enhance the quality of care and reduce the disease burden.
